# TIPE2 acts as a biomarker for tumor aggressiveness and suppresses cell invasiveness in papillary thyroid cancer (PTC)

**DOI:** 10.1186/s13578-018-0247-x

**Published:** 2018-08-31

**Authors:** Wenyu Jia, Zequn Li, Junyu Chen, Lei Sun, Chuanqian Liu, Shaping Wang, Jingwei Chi, Jun Niu, Hong Lai

**Affiliations:** 1 Department of Endocrinology, Shandong Provincial Hospital Affiliated to Shandong University, Jinan, Shandong People’s Republic of China; 2 Institute of Endocrinology and Metabolism, Shandong Academy of Clinical Medicine, Jinan, Shandong People’s Republic of China; 3 Department of General Surgery, Affiliated Hospital of Qingdao University, Qingdao, Shandong People’s Republic of China; 4 Department of Endocrinology, Qilu Hospital of Shandong University, 107# Wenhua Xi Road, Jinan, 250012 Shandong People’s Republic of China; 5 Department of Traditional Chinese Medicine, The First People’s Hospital of Jining, Jining, Shandong People’s Republic of China; 6 Clinical Laboratory, Weihai Wendeng Central Hospital, Weihai, Shandong People’s Republic of China; 7 Key Laboratory of Thyroid Disease, Affiliated Hospital of Qingdao University, Qingdao, Shandong People’s Republic of China; 8 Department of General Surgery, Qilu Hospital of Shandong University, Jinan, Shandong People’s Republic of China

**Keywords:** TIPE2, PTC, Invasiveness, Rac1, uPA, MMP9

## Abstract

**Background:**

Tumor necrosis factor (TNF)-alpha-induced protein 8-like 2 (TIPE2 or TNFAIP8L2) is a newly described negative immune regulator and is closely associated with various tumors. However, the expression and roles of TIPE2 in PTC is unknown.

**Results:**

In the present study, TIPE2 upregulation in PTC tissues was found to be negatively associated with tumor size, capsule infiltration, peripheral infiltration and tumor T stage, which could be used to predict tumor invasiveness. TIPE2 overexpression significantly suppressed the viability, proliferation, migration and invasion of PTC cells. Moreover, TIPE2 suppressed tumor invasiveness by inhibiting Rac1, leading to decreased expression of uPA and MMP9.

**Conclusions:**

These results indicate that TIPE2 is a potential biomarker for predicting tumor aggressiveness and suppresses tumor invasiveness in a Rac1-dependent manner.

**Electronic supplementary material:**

The online version of this article (10.1186/s13578-018-0247-x) contains supplementary material, which is available to authorized users.

## Background

Thyroid cancer is the most common malignancy of the endocrine system and its incidence has been rapidly growing worldwide over the past few decades. Despite equivalent death rates, thyroid cancer incidence rates are 3 times higher in women than in men [1, 2]. Thyroid cancer could be categorized into four histologic types: papillary, follicular, medullary, and anaplastic. Papillary thyroid cancer (PTC) accounts for the majority of cases [3]. The standard therapies of PTC include radical excision, radio-iodine treatment, and thyroid stimulating (TSH) hormone suppression. Although the prognosis of PTCs is generally good, recurrence occurs in patients with aggressive tumors, which usually leads to a poor prognosis [4–7]. Therefore, a better understanding of the molecular mechanisms that are associated with the aggressiveness of PTC is needed for improving the prognosis of the patients.

Tumor necrosis factor-α-induced protein-8 (TNFAIP8)-like-2 (TIPE2) is a newly described member of the TNFAIP8 family. The family contains four members, TNFAIP8, TIPE1, TIPE2 and TIPE3 [8, 9]. TIPE2 was initially discovered as an immune negative regulator that is involved in both innate and adaptive immunity. It was first isolated from the inflamed spinal cord of experimental autoimmune encephalomyelitis (EAE) mice and its deficiency leads to lethal multi-organ inflammation [8, 10, 11]. TIPE2 is preferentially expressed in lymphoid tissues and a variety of specific epithelial tissues such as glandular epithelium and squamous epithelium [12, 13]. Interestingly, it has been demonstrated that TIPE2 also plays a pivotal role in tumorigenesis and tumor development in the recent years. TIPE2 has been found to be a tumor suppressor in a number of tumors [14–17]. However, detailed mechanisms were still required in most of these studies.

Our previous research demonstrated that by directly binding with Rac1-GTPases, murine TIPE2 dictates the strength of phagocytosis and oxidative burst in innate immunity, while human TIPE2 could inhibit various malignant behaviors of tumor cells and suppress tumor metastasis [14, 16, 18]. Rac1 is a member of the Rho family of small GTPases. It has been implicated in a wide variety of cellular processes, including cytoskeletal reorganization, gene transcription, and cell migration [19–21]. Rac1 is highly expressed in a number of cancer cell lines and plays an important role in regulating tumor progression [22, 23]. It has been demonstrated that Rac1 regulates various downstream effector molecules related to tumor aggressiveness, such as MMP9 and uPA [14, 24]. Research has found that Rac1 is overexpressed in PTC and is responsible for activating various oncogenic signaling [25, 26].

In the present study, we demonstrated for the first time that TIPE2 is overexpressed in PTC tissues. Interestingly, it is negatively associated with tumor aggressiveness as its level is lower in infiltrated tissue than in non-infiltrated tissue. Further analysis also demonstrated that TIPE2 acts as a biomarker for the diagnosis of PTC and evaluation of tumor invasiveness. Our results also indicate that TIPE2 is crucial in the malignant progress of PTC. Moreover, TIPE2 serves as a tumor suppressor via inhibiting the activation of Rac1. All these results suggest that TIPE2 is a key regulator during the progress of PTC, which deserves to be further explored.

## Methods

### Tissue samples

A total of 89 human PTC specimens and the related adjacent normal tissues were collected from Qilu Hospital of Shandong University. Clinicopathological classification and staging were determined according to the American Joint Committee on Cancer (AJCC) criteria [27]. All samples were fixed in 40 g/L formaldehyde and were embedded in paraffin for histological diagnosis and immunohistochemical analysis. The study was approved by the Institutional Review Board of Shandong University, China. Written informed consent was obtained from all subjects.

### Immunohistochemistry (IHC)

Immunohistochemistry was performed using paraffin-embedded tissue sections. The sections were dewaxed and hydrated, followed by antigen retrieval (in 0.01 mol/L citrate buffer solution, pH 6.0, heated to boil for 2–3 min in a stainless steel pressure cooker). Endogenous peroxidase was blocked using a 3% hydrogen peroxide solution. The sections were incubated with the blocking goat serum for 15 min, then immunostained with rabbit antibody against TIPE2 (dilution 1:100, Abcam, UK) at 4 °C overnight. Secondary staining was performed with HRP-conjugated anti-rabbit IgG using a MaxVsion Kit and a 3, 5-diaminobenzidine (DAB) peroxidase substrate kit (Maixin Co, Fuzhou, China). The sections were counterstained with hematoxylin.

### Evaluation of immunohistochemical staining

The immunohistochemical staining was independently evaluated by two experienced pathologists in a blinded manner. Staining was semi-quantitatively scored based on both the staining intensity (0, negative; 1, weak; 2, moderate; 3, strong) and the percentage of positively stained cells (0, 0%; 1, 1–25%; 2, 26–50%; 3, 51–75%; 4, 76–100%). The two scores for each specimen were then combined to come up with a final TIPE2 expression score. The cut-off point of the sum of the scores were defined as follows: 0–3, low expression; 4–7, high expression. The appropriateness of the cut-off point was validated by ROC analysis.

### Cell culture

The human PTC cell lines, TPC-1 and B-CPAP, obtained from the American Type Culture Collection (ATCC), were separately maintained in DMEM and RPMI 1640 medium (Gibco, CA, USA) supplemented with 10% inactivated fetal bovine serum (FBS) (Gibco) in a humidified cell incubator with an atmosphere of 5% CO_2_ at 37 °C.

### Plasmid construction and transfection

The wild type TIPE2 plasmid was generated from the cDNA clone by PCR and cloned in frame with a C-terminal Flag into the PRK5 vector. The mutant TIPE2 plasmid in which the TIPE2 N-terminal lysine or arginine residues, Lys-15, Lys-16, and Arg-24 were replaced with glutamine or alanine was generated by PCR-based site-directed mutagenesis as previously described [18]. The siRNA used in the research were purchased from Sigma-Aldrich (Louis, USA). Related sequences were as follows. Rac1-siRNA: forward 5′-GCAAACAGAUGUGUUCUUA-3′, reverse 5′-UAAGAACACAUCUGUUUGC-3′. The negative control siRNA sequence: forward 5′-UUCUCCGAACGUGUCACGUTT-3′, reverse 5′-ACGUGACACGUUCGGAGAATT-3′. Transfection of PTC cells with plasmid or siRNA was performed using Lipofectamine 2000 according to the manufacturer’s protocols (Invitrogen, Carlsbad, CA, USA).

### RNA isolation and real-time quantitative PCR

Total RNAs were extracted from transfected cells using TRIzol reagent (Invitrogen, Carlsbad, CA, USA) and were reverse transcribed into cDNA using a Rever Tra Ace qPCR Kit (Toyobo, Osaka, Japan). Real-time quantitative PCR was performed using an UltraSYBR Mixture (CWBIO). The sequences of the sense and antisense primers were as follows: TIPE2: 5′-ACTGA GTAAGATGGCGGGTCG-3′, and 5-TTCTGGCGAA AGCGGGTAG-3′; Rac1: 5′-AT GTCCGTGCAAAGTGGTATC-3′, and 5-CTCGGATCGCTTCGTCAAACA-3′; GAPDH: 5′-AACGGATTTGGTCGTATTGGG-3′, and 5′-CCTGGA AGATGGTGAT GGGAT-3′; MMP9: 5-GCATTCAGGGAGACGCCCATTT AACGACA-3′, and 5′-CTGACACTCCCGGTGGG AAATCA-3′; uPA: 5′-ACTACATTGTCTACCTGGGTCGGTC-3′ and 5′-ATGCAA GATGAGTTGCTCCACTTC-3′. Relative gene expression levels were normalized to GAPDH as control.

### Cell viability assays

A total of 3000 cells were seeded in 96-well plates in triplicate wells and cultured for the indicated times. Cell viability assay was performed and evaluated using Cell Counting Kit-8 (CCK8) (Beyotime, Haimen, China). The absorbance was determined at 450 nm. Each time point was replicated in three wells, and the experiment was independently performed at least three times.

### Western blot

After 48 h of transfection, PTC cells were subjected to protein extraction using cell lysis buffer containing 1% protease inhibitors. Protein concentrations of the homogenized lysates were measured using a BCA protein assay kit (Sangon, Shanghai, China). Aliquots containing 30 μg of protein were separated by 10% SDS-PAGE and then transferred to PVDF membranes (Millipore, Billerica, MA, USA). Membranes were incubated overnight at 4 °C with the following primary antibodies: mouse monoclonal antibody against TIPE2 (1:300; Abcam, UK), or anti-β-actin (1:1000; ZSGB-Bio, Beijing, China), followed by secondary antibodies (1:2000; goat-anti rabbit or mouse IgG, ZSGB-Bio) conjugated to peroxidase for 1 h at room temperature. After washing, immunoreactivity was visualized using an enhanced chemiluminescence kit (Millipore, Billerica, MA, USA) according to the manufacturer’s instructions.

### Cell proliferation assays

Cell proliferation assays were performed using MTT (3-(4,5-dimethylthiazol-2-yl)-2,5-diphenyl tetrazolium bromide). A total number of 3000 cells were seeded in 96-well plates and incubated for 24 h after TIPE2 overexpression, MTT at 5 mg/ml was added and incubated for 4 h. Finally, the medium was aspirated, DMSO was added, and the absorbance was determined at 490 nm. Each time point was repeated in three wells, and the experiment was independently performed at least three times.

### Transwell assays for cell migration and invasion

Tumor cell migration assays were analyzed in 24-well Boyden chambers with 8-μm pore size polycarbonate membranes (Costar, Acton, USA). For invasion assays, the membranes were precoated with 50 μg Matrigel (BD Biosciences, San Diego, USA) to simulate matrix barriers. Then, 1 × 10^5^ cells were resuspended in 200 μl serum-free medium and seeded into the upper chamber, and the lower compartments were filled with 600 μl medium with 10% FBS. After 12 h incubation for migration and 24 h incubation for invasion assay, the upper surface of the Transwell membrane was wiped gently with a cotton swab to remove the non-migrating cells. The membranes were fixed with methanol and stained with 0.1% crystal violet for 20 min. The stained cells were counted under a light microscope at 200× magnification in at least five fields. Specific Rac1 inhibitor NSC23766 (Calbiochem, San Diego, USA) was used to inhibit Rac1 activity in several experiments.

### MMP activity assays

A total of 2.5 × 10^5^ cells were cultured in a six-well culture plate and then transfected with TIPE2 overexpression plasmid for 24 h. The levels of secreted MMP-9 in the culture supernatant were determined using an enzyme-linked immunosorbent assay (ELISA) following the manufacturer’s ELISA kit guidelines (R&D, USA). Samples were assayed in triplicate and calibrated against a standard curve.

### Statistical analysis

The associations between TIPE2 expression and clinicopathological parameters were analyzed by the Chi square test and Fisher’s exact test. Quantitative data are presented as the mean ± SD. The statistical significance was determined by two-tailed paired Student’s t-test in two groups and one-way ANOVA in multiple groups. Receiver operating characteristic (ROC) curve analysis was performed to assess the diagnostic value of TIPE2 in PTC. All statistical analyses were performed using SPSS 18.0 software (SPSS Inc., Chicago, USA), and a *P* value < 0.05 was considered statistically significant.

## Results

### TIPE2 protein expression was associated with tumor invasiveness in PTC

**Fig. 1:**
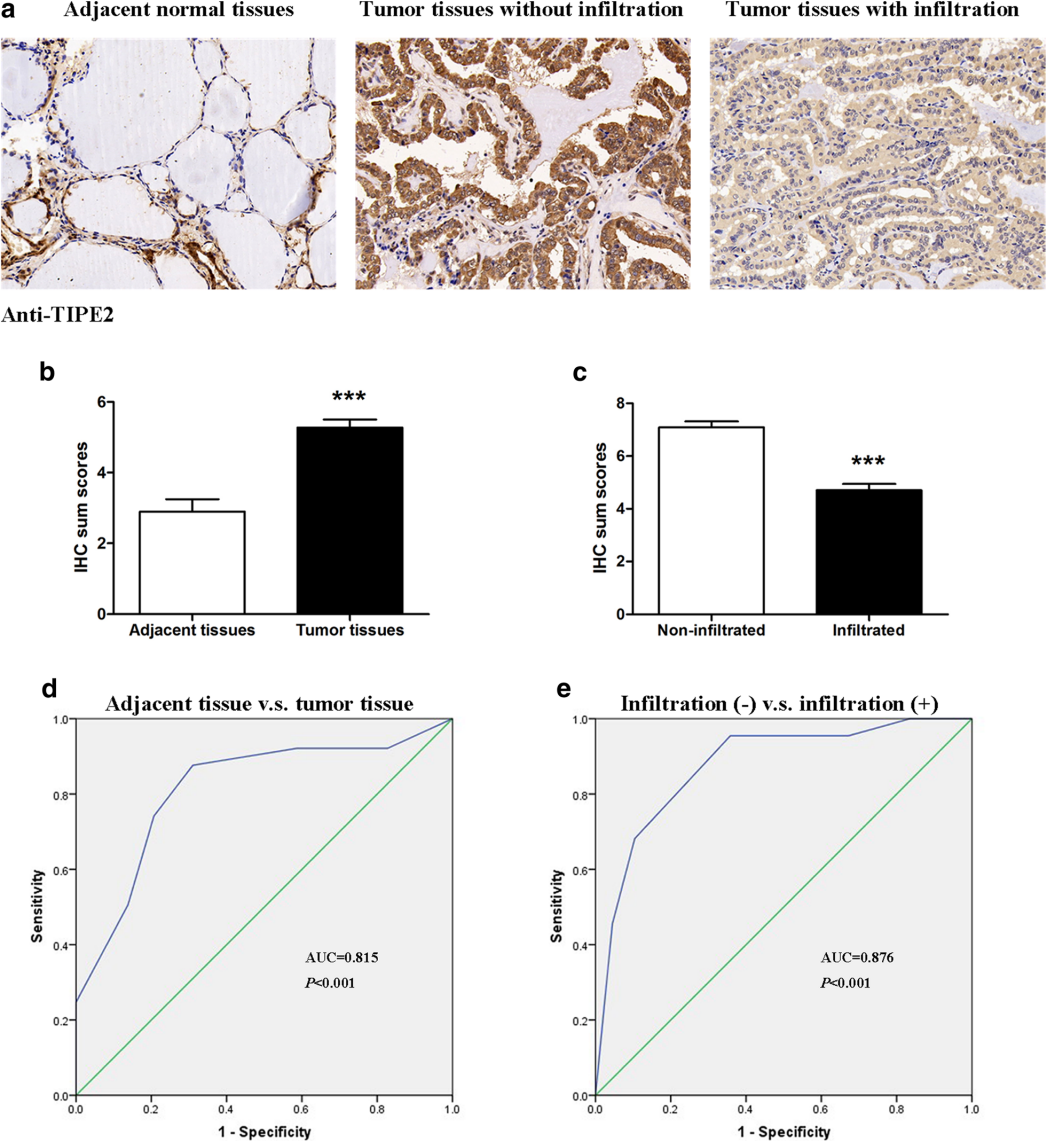
The expression and significance of TIPE2 in normal thyroid tissues and PTC tissues. **a** IHC results (×200 magnification) of TIPE2 expression in normal thyroid tissues and PTC tissues with or without infiltration. **b** IHC sum scores were used to compare TIPE2 expression in normal thyroid tissues and PTC tissues. **c** IHC sum scores were used to compare TIPE2 expression in PTC tissues with or without infiltration. ***P < 0.001. **d** The ROC curve showed clear separation between normal thyroid tissues and PTC tissues, with an AUC of 0.815 (*P *< 0.001). **e** The ROC curve was established and showed strong separation between patients with and without tumor infiltration, with an AUC of 0.876 (*P *< 0.001)

To explicit the expression condition of TIPE2 in PTC tissues, we conducted an IHC analysis using a total number of 89 paraffin-embedded PTC tissues and corresponding adjacent normal tissues. Results showed that TIPE2 staining was low in adjacent normal tissues, while strong TIPE2 expression was observed in tumor tissues. Moreover, the expression of TIPE2 decreased in PTC tissues with infiltration (Fig. 1a–c). To further explore the clinical significance of TIPE2 expression in PTC tissues, we analyzed the correlation between TIPE2 expression and various clinicopathological factors. As shown in Table 1, TIPE2 expression was negatively associated with tumor size (*P *= 0.0002), capsule infiltration (*P *= 0.0085), peripheral infiltration (*P *= 0.0036) and tumor T stage (*P *= 0.0001), while no significant association was shown between TIPE2 expression and age (*P *= 0.3403), gender (*P *= 0.3841) and lymph node metastasis (*P *= 0.0941). All these results indicated that TIPE2 protein expression was associated with tumor invasiveness in PTC and TIPE2 may be involved in the progression of PTC.

**Table 1:** Correlations between TIPE2 expression and clinicopathological characteristics in PTC

Variable	Number	TIPE2 expression		P value
		Low	High	
Number of patients	89	23	66	
Age (years)				
< 45	50	15	35	0.3403
≥ 45	39	8	31	
Gender				
Male	20	7	13	0.3841
Female	69	16	53	
Tumor size (cm)				
≤ 1	27	4	23	*0.0002*
1.5–4	57	12	45	
> 4	5	5	0	
Capsule infiltration				
Negative	22	1	21	*0.0085*
Positive	67	22	45	
Peripheral infiltration				
Negative	75	15	60	*0.0036*
Positive	14	8	6	
T stage				
T1	65	8	57	*0.0001*
T2–T4	24	15	9	
Lymph node metastasis				
Negative	45	8	37	0.0941
Positive	44	15	29	

The bold emphasis means the results have significant difference

### TIPE2 was a promising biomarker for diagnosis of PTC and prediction of tumor infiltration

It has been previously demonstrated that TIPE2 protein was up-regulated in PTC tissues compared with normal tissues. To determine the diagnostic value of TIPE2 expression in PTC, we constructed receiver operator characteristic (ROC) curves and calculated the area under the curve (AUC) to access whether TIPE2 expression was able to differentiate tumor tissues and normal tissues. The ROC curves showed that the AUC for TIPE2 in discriminating PTC tissues and normal tissues was up to 0.815 (Fig. 1d, CI (95%) 0.733–0.880, *P *< 0.001), with an estimated sensitivity and specificity of 87.64 and 68.97%, respectively (Additional file 1: Table S1).

As TIPE2 expression was decreased in aggressive PTC tissues. The predictive accuracy of TIPE2 expression to distinguish between patients with and without tumor infiltration was accessed using ROC curve. The AUC value for predicting tumor infiltration in PTC was 0.876 (Fig. 1e, CI (95%) 0.789–0.936, *P *< 0.001), with an estimated sensitivity and specificity of 95.45 and 64.18%, respectively (Additional file 1: Table S1). All these results demonstrated that TIPE2 could serve as a promising biomarker for the diagnosis of PTC and the prediction of tumor invasiveness.

### TIPE2 overexpression markedly suppressed the viability, proliferation, migration and invasion of PTC cells

**Fig. 2:**
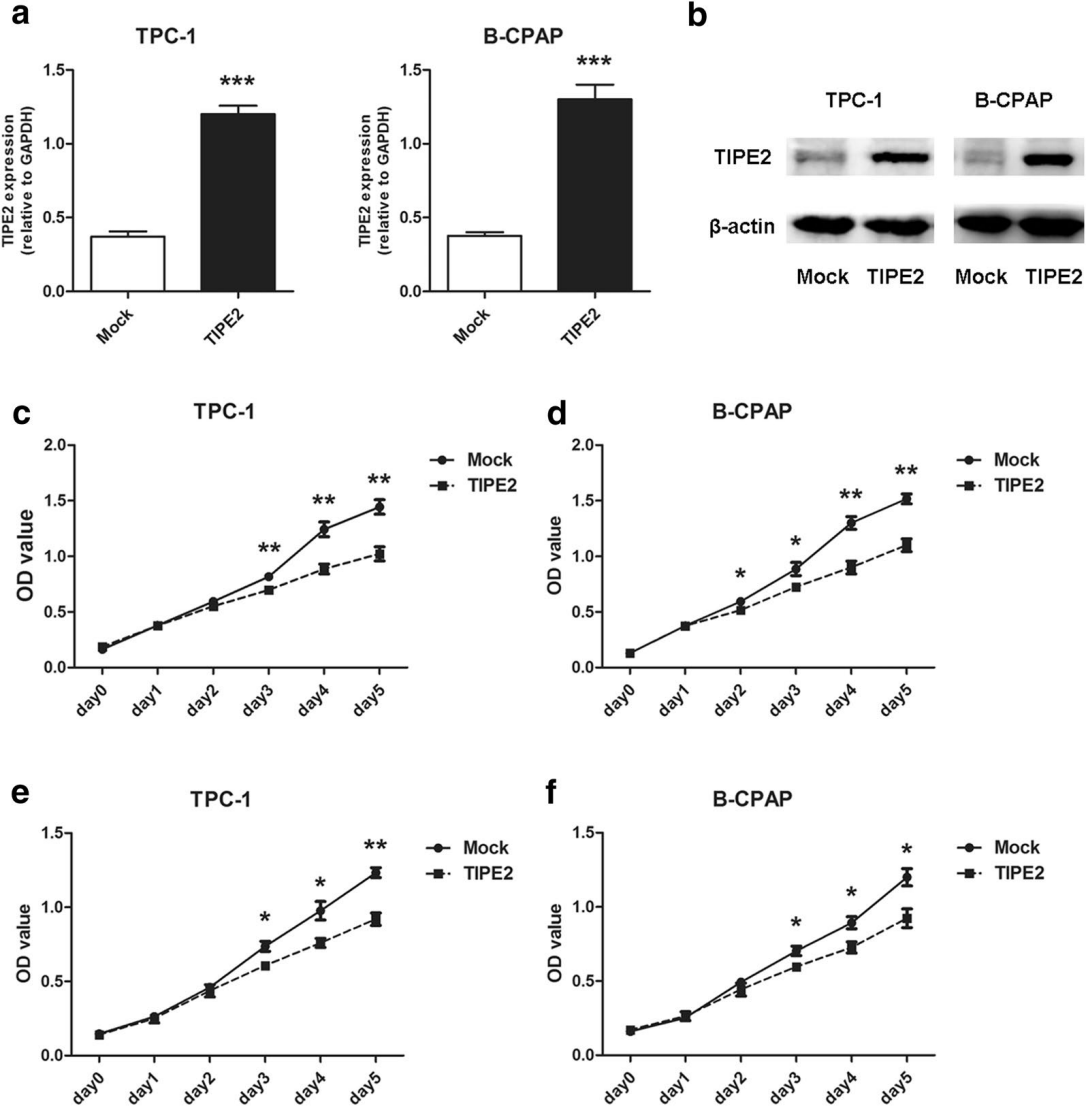
TIPE2 overexpression markedly suppressed the viability and proliferation of PTC cells. A-B. Real-time PCR (**a**) and western-blot (**b**) showed that TIPE2 expression was significantly upregulated after transfection of the PRK5-TIPE2 plasmid in TPC-1 and B-CPAP cells. **c**, **d** After TIPE2 overexpression, CCK8 assays were conducted to evaluate the viability of TPC-1 (**c**) and B-CPAP cells (**d**). **e**, **f** MTT assays were conducted to evaluate the proliferation of TPC-1 (**e**) and B-CPAP cells (**f**) after TIPE2 overexpression. Data represent the mean ± SD of three independent experiments. *P < 0.05; **P < 0.01; ***P < 0.001

To further elucidate the roles of TIPE2 in PTC, we explored the effect of TIPE2 on various of malignant biological behaviors of PTC cells. First, the expression of TIPE2 in human PTC cell lines, TPC-1 and B-CPAP was detected by real-time PCR, results revealed a low TIPE2 expression in these two cell lines. Therefore, we overexpressed TIPE2 in the two cell lines by the transfection of PRK5-TIPE2 recombinant plasmid. As is shown in Fig. 2a, b, TIPE2 expression was significantly increased. CCK8 assays were conducted to evaluate the effect of TIPE2 on the viability of PTC cells. The results showed that TIPE2 overexpression in TPC-1 and B-CPAP cells markedly suppressed cell viability (Fig. 2c, d). Then MTT assays were performed to investigate the effect of TIPE2 on the proliferation of PTC cells. The results showed that TIPE2 overexpression was significantly associated with decreased cell proliferation (Fig. 2e, f).

**Fig. 3:**
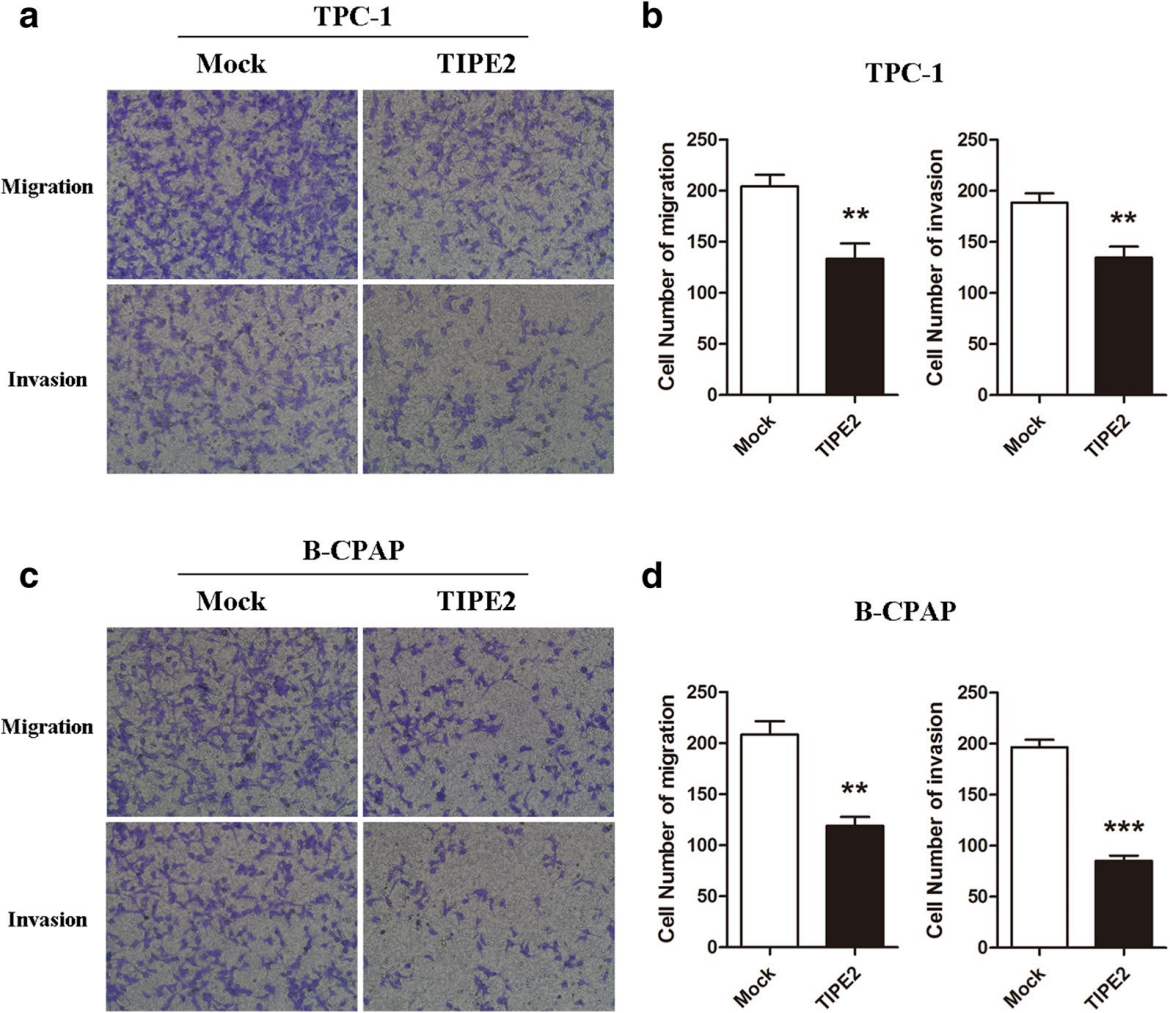
TIPE2 notably suppressed the migration and invasion of PTC cells. **a** TPC-1 cells transfected with the TIPE2 overexpression plasmid were used for Transwell migration and invasion assays. **b** Statistic results were shown by counting five fields of TPC-1 cells in the lower chambers (×200 magnification). **c** B-CPAP cells transfected with the TIPE2 overexpression plasmid were used for Transwell migration and invasion assays. **d** Statistic results were shown by counting five fields of B-CPAP cells in the lower chambers (×200 magnification). Data represent the mean ± SD of three independent experiments. **P < 0.01; ***P < 0.001

As it has been demonstrated that TIPE2 expression is negatively correlated with tumor invasiveness. We performed Transwell migration and invasion assays in PTC cells after TIPE2 overexpression. As TIPE2 had no effect on cell viability and proliferation within the 12-h period, which eliminated the potential confounding influence of TIPE2 induced cell viability or proliferation suppression on cell migration and invasion. The results indicated that TIPE2 overexpression significantly inhibited the migration and invasion capacities of both TPC-1 and B-CPAP cells (Fig. 3a–d).

Taken together, these data suggested that TIPE2 acts as a tumor suppressor by suppressing the viability, proliferation, migration and invasion of PTC cells.

### TIPE2 suppressed the invasiveness of PTC cells by inhibiting Rac1

**Fig. 4:**
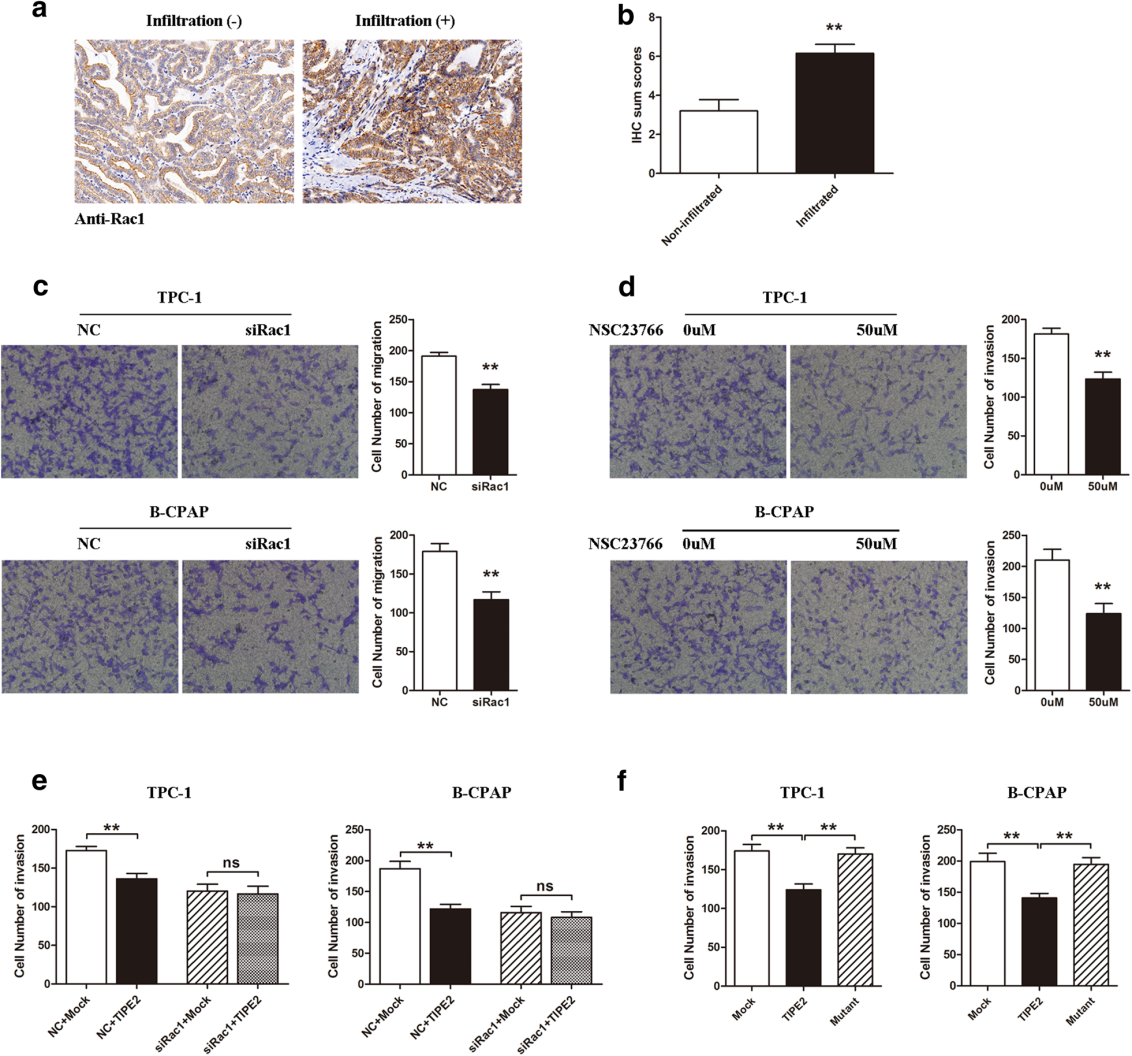
TIPE2 suppressed the invasiveness of PTC cells via inhibiting Rac1. **a** IHC results (×200 magnification) of Rac1 expression in PTC tissues with or without infiltration. **b** IHC sum scores were used to compare TIPE2 expression in PTC tissues with or without infiltration. **c** The invasiveness of TPC-1 and B-CPAP cells were measured by Transwell invasion assays after transfection of specific Rac1 siRNA. **d** After treatment of NSC23766, Transwell invasion assays were used to evaluate the invasiveness of TPC-1 and B-CPAP cells. **e** TPC-1 and B-CPAP cells co-transfected with Rac1-specific siRNA and TIPE2 overexpressing plasmid were used for Transwell invasion assays. **f** TPC-1 and B-CPAP cells that transfected with Mock, wild type TIPE2 and mutant TIPE2 plasmids were used for Transwell invasion assays. Data are shown as the mean ± SD, and the results shown are representative of 3 independent experiments. Five fields of cells in the lower compartment were counted in Transwell assays (200 × magnification). **P < 0.01

It has been demonstrated that Rac1 is a key molecule in regulating the invasiveness of PTC, and our previous research found that TIPE2 functions through inhibiting Rac1 in hepatocellular carcinoma (HCC) and non-small cell lung cancer (NSCLC) [14, 16]. Here we hypothesized that TIPE2 may suppress the invasiveness by inhibiting Rac1 in PTC. To confirm our hypothesis, we performed the following experiments. First, we detected Rac1 expression in PTC tissues by IHC, and we found that Rac1 expression was markedly up-regulated in PTC tissues with infiltration compared with that in PTC tissues without infiltration (Fig. 4a, b). Then, using both specific Rac1 silencing RNA and NSC23766, a Rac1 inhibitor, we investigated the effect of Rac1 on the invasiveness of PTC cells. We demonstrated that both Rac1 silencing and Rac1 activity inhibition effectively suppressed the invasiveness of PTC cells (Fig. 4c, d). Moreover, by co-transfecting Rac1 siRNA and TIPE2 overexpressing plasmid, we demonstrated that Rac1 silencing eliminated the inhibitory effect of TIPE2 on the invasiveness of PTC cells (Fig. 4e). Furthermore, the mutation of TIPE2 in sites which binds to Rac1, reversed this inhibitory effect on tumor cell invasiveness (Fig. 4f). All these results demonstrated that TIPE2 suppressed the invasiveness of TPC cells by inhibiting Rac1.

### TIPE2 suppressed Rac1 downstream effectors, uPA and MMP9 expression in PTC cells

**Fig. 5:**
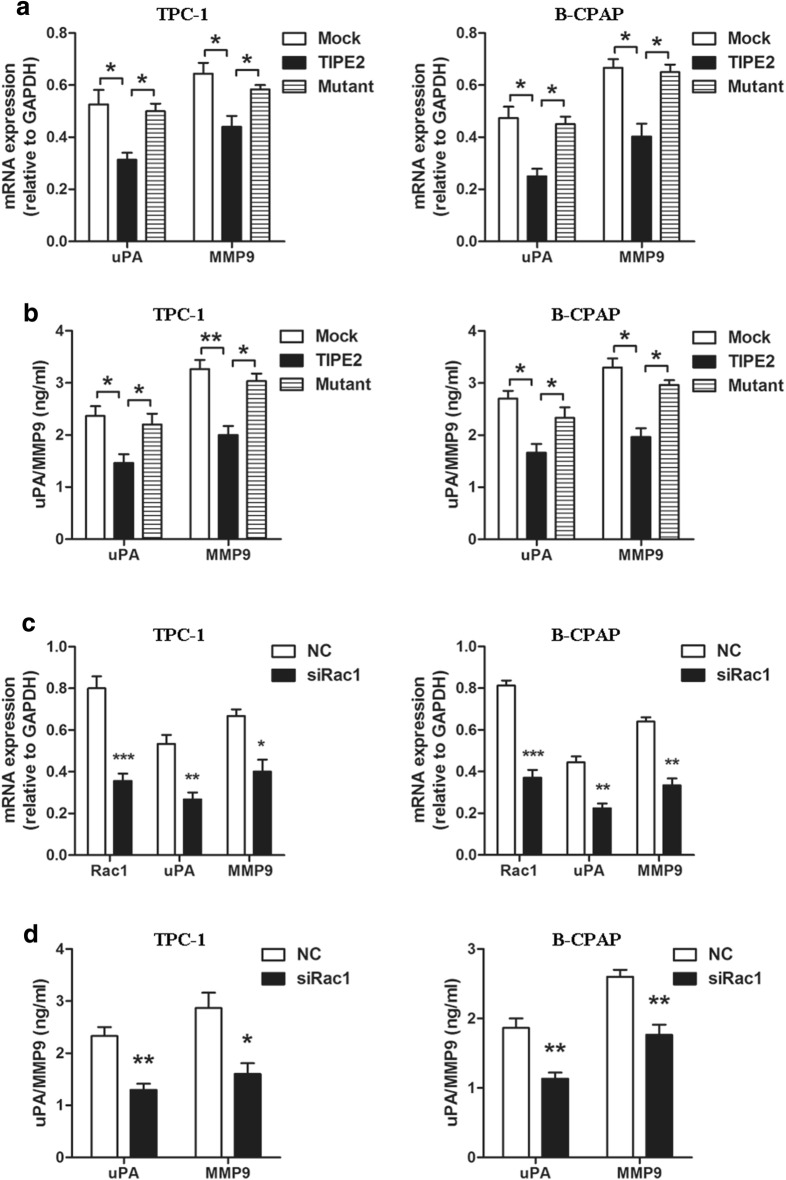
TIPE2 suppressed uPA and MMP9 expression via inhibiting Rac1 in PTC cells. **a** The expression of uPA and MMP9 at mRNA level were detected by real-time PCR after transfection with Mock, wild type TIPE2 and mutant TIPE2 plasmids in PTC cells. **b** The protein level of uPA and MMP9 were detected by ELISA in PTC cells after transfected with Mock, wild type TIPE2 and mutant TIPE2 plasmids. **c** The expression of uPA and MMP9 at mRNA level were detected by real-time PCR in TPC-1 and B-CPAP cells after Rac1 silencing. **d** The protein level of uPA and MMP9 were detected by ELISA in TPC-1 and B-CPAP cells after Rac1 silencing

It is known that the uPA and MMP9 are important Rac1 downstream effectors that are responsible for degrading the extracellular matrix components, which is essential for the invasiveness of tumor cells [28–30]. Therefore, we investigated the effect of TIPE2 on uPA and MMP9 expression in PTC cells. As is shown in Fig. 5a, b, TIPE2 significantly decreased uPA and MMP9 expression at both the mRNA and protein levels, while TIPE2 mutation reversed this inhibitory effect. Moreover, we found that the expression of uPA and MMP9 also decreased after Rac1 silencing at both the mRNA and protein levels (Fig. 5c, d). These results indicated that TIPE2 decreased uPA and MMP9 expression via inhibiting Rac1.

## Discussion

The incidence of PTC has been rapidly growing over the past few decades. Although the prognosis of PTCs is generally good, tumor invasiveness is one of the major risk factor that leads to poor prognosis [1, 3, 31]. Therefore, elucidating the mechanisms underlying the invasiveness of PTC may facilitate the development of clinical strategies. In the present study, we found that TIPE2 expression was significantly associated with tumor invasiveness. Moreover, we demonstrated that TIPE2 suppressed the migratory and invasive capacities of PTC by inhibiting Rac1, indicating that forced TIPE2 expression might be a novel strategy for the treatment of PTC.

Although TIPE2 was primarily identified as an immune regulator, it has been demonstrated that TIPE2 also expressed in various non-immune tissues such as glandular epithelium, squamous epithelium [12, 13]. Decreased TIPE2 expression were previously reported in a number of tumors such as hepatocellular carcinoma (HCC), gastric cancer, glioma and prostate cancer [14, 15, 17, 32]. However, increased TIPE2 expression was also observed in some specific tumors including colorectal cancer (CRC), non-small cell lung cancer (NSCLC) and skin squamous cancer [16, 33, 34]. This study also demonstrated that TIPE2 expression was up-regulated in PTC compared with the adjacent normal thyroid tissue. This expression may be due to the specific expression characteristics of TIPE2. As TIPE2 is preferably expressed in glandular epithelium, and according to previous research, TIPE2 is negatively expressed in normal thyroid gland [12, 13]. We deduced that increased TIPE2 expression resulted from the glandular metaplasia. The expression of TIPE2 in CRC, NSCLC and skin squamous carcinoma verified our hypothesis. Histologically, most of the CRC tissues are glandular carcinoma, glandular carcinoma and squamous carcinoma account for the majority of all NSCLCs. Further analysis revealed that TIPE2 expression decreased with tumor progression, and reduced TIPE2 expression was significantly associated with tumor size, capsule infiltration, peripheral infiltration and T stage. Moreover, ROC analysis indicated that TIPE2 expression had discriminative validity in differentiating tumor tissues and tumor invasiveness. All these results suggested that TIPE2 may serve as a potential immunohistochemical marker, and TIPE2 staining in the surgical specimens could be used to evaluate the risks of tumor invasiveness.

TIPE2 mainly acts as a tumor suppressor by suppressing tumor proliferation, migration, invasion and angiogenesis in a variety of tumors [14–16]. However, the role of TIPE2 in PTC has not been elucidated. Our results showed that TIPE2 overexpression suppressed the proliferation, migration and invasion of PTC cells significantly. Therefore, our data indicated that although TIPE2 expression was up-regulated in PTC tissues, TIPE2 still serves as a tumor suppressor in PTC, which is similar to that in NSCLC. The decreased TIPE2 expression in more aggressive PTC tissues also verified our conclusion.

The detailed mechanism of how TIPE2 suppressed tumor aggressiveness remains largely unknown. It has been demonstrated that TIPE2 may function as a tumor suppressor by inhibiting several classic oncogenic signaling pathways [15, 17]. Our previous research demonstrated that TIPE2 suppressed the migration and invasion of HCC and NSCLC cells by inhibiting Rac1 [14, 16]. Rac1 is a member of the Rho family of small GTPases and has been implicated in a wide variety of cellular processes, especially in cell motility [19, 23, 35]. Research found that Rac1 plays a pivotal role in the aggressiveness of PTC [25, 26, 36]. To determine whether TIPE2 functions through inhibiting Rac1, we generated a mutant TIPE2 plasmid, which lost the Rac1 binding site. In addition, specific Rac1 inhibitor and siRNA were also used. Results indicated that Rac1 is a promising target of TIPE2 in PTC. Previous studies showed that uPA and MMPs, which further were responsible for degrading extracellular matrix (ECM), played important roles in the aggressiveness of PTC. Moreover, uPA and MMPs have been demonstrated to be crucial downstream effectors of Rac1 [37–39]. Here we demonstrated that TIPE2 suppressed tumor aggressiveness via inhibiting Rac1, which subsequently decreased the expression of uPA and MMP9 in PTC cells.

There were several limitations in our research. The specificity of TIPE2 in predicting tumor progression was lower than 70%. Therefore, an increased sample size is needed to further determine the precise predictive effect of TIPE2 in PTC progression. In addition, the correlation between TIPE2 expression and the prognosis of PTC patients still needs to be further investigated.

The aggressiveness of PTC remains the main reason for the poor prognosis of the disease. In the present study, we demonstrated that TIPE2 serves as an ideal immunohistochemical biomarker for the differentiation of tumor aggressiveness in PTC. Moreover, TIPE2 acts as a tumor suppressor by inhibiting Rac1 related signaling. Taken together, we hypothesize that TIPE2 expression was up-regulated during the tumorigenesis of PTC, and the expression of TIPE2 decreased accompanied with increased aggressiveness of tumor cells. In tumor tissues with high TIPE2 expression, the invasiveness of tumor cells might be suppressed by TIPE2. Moreover, TIPE2 is an endogenous inhibitor of Rac1, forced TIPE2 expression may present a potential strategy for the treatment of patients with aggressive PTC.

## Conclusions

Human negative immune regulator TIPE2 was up-regulated in PTC tissues, which was found to be negatively associated with tumor size, capsule infiltration, peripheral infiltration and tumor T stage. The expression of TIPE2 serves as an IHC biomarker for the evaluation of tumor aggressiveness. TIPE2 suppressed a number of malignant behaviors of tumor cells, such as viability, proliferation, migration and invasion. Mechanically, TIPE2 suppressed tumor invasiveness by inhibiting Rac1, which subsequently decreased the expression of uPA and MMP9. All these results indicate that TIPE2 is a potential biomarker for predicting tumor aggressiveness and suppresses tumor invasiveness in a Rac1-dependent manner in PTC.

## Additional file

**Additional file 1: Table S1.** Sensitivity, specificity, and positive and negative predictive values for the detection of PTC and tumor infiltration using TIPE2 expression.

## Abbreviations

TIPE2: tumor necrosis factor (TNF)-alpha-induced protein 8-like 2
PTC: papillary thyroid cancer
TSH: thyroid stimulating hormone
EAE: experimental autoimmune encephalomyelitis
AJCC: the American Joint Committee on Cancer
IHC: immunohistochemistry
AUC: area under the curve
HCC: hepatocellular carcinoma
NSCLC: non-small cell lung cancer
CRC: colorectal cancer
ECM: extracellular matrix
