# Supplementary material for: TIPE2 acts as a biomarker for tumor aggressiveness and suppresses cell invasiveness in papillary thyroid cancer (PTC)
Source: Cell Biosci. 2018 Aug 31;8:49. doi: 10.1186/s13578-018-0247-x (PMC6119276; doi:10.1186/s13578-018-0247-x)
Supplement: Supplementary file 1 — Additional file 1: Table S1. Sensitivity, specificity, and positive and negative predictive values for the detection of PTC and tumor infiltration using TIPE2 expression. [file 13578_2018_247_MOESM1_ESM.doc]

| **Additional file 1: Table S1: Sensitivity, Specificity, and Positive and Negative Predictive Values for the detection of PTC and tumor infiltration using TIPE2 expression** | | | | | | | | | |
| --- | --- | --- | --- | --- | --- | --- | --- | --- | --- |
| **IHC scores** | **Sensitivity** | **95% CI** | **Specificity** | **95% CI** | **PPV^a^** | **95% CI** | **NPV^b^** | **95% CI** | **Significance** |
| >3 | 87.64 | 79.0 - 93.7 | 68.97 | 49.2 - 84.7 | 89.7 | 81.3 - 95.2 | 64.5 | 45.4 - 80.8 | PTC detection |
| <5 | 95.45 | 77.2 - 99.9 | 64.18 | 51.5 - 75.5 | 46.7 | 31.7 - 62.1 | 97.7 | 88.0 - 99.9 | Tumor infiltration detection |

**^a^PPV= Positive Predictive Value**

**^b^NPV= Negative Predictive Value**
